# The expanding network of mineral chemistry throughout earth history reveals global shifts in crustal chemistry during the Proterozoic

**DOI:** 10.1038/s41598-022-08650-x

**Published:** 2022-03-23

**Authors:** Eli K. Moore, Josh J. Golden, Shaunna M. Morrison, Jihua Hao, Stephanie J. Spielman

**Affiliations:** 1 Department of Environmental Science, School of Earth and the Environment, Rowan University, Glassboro, NJ USA; 2 Department of Geosciences, University of Arizona, Tucson, AZ USA; 3 Earth and Planets Laboratory, Carnegie Institution for Science, Washington, DC USA; 4 CAS Key Laboratory of Crust-Mantle Materials and Environments, School of Earth and Space Sciences, University of Science and Technology of China, Hefei, 230026 China; 5 CAS Center for Excellence in Comparative Planetology, USTC, Hefei, 230026 Anhui China; 6 Department of Marine and Coastal Sciences, Rutgers University, New Brunswick, NJ USA; 7 Department of Biological Sciences, College of Science and Mathematics, Rowan University, Glassboro, NJ USA

**Keywords:** Planetary science, Geochemistry, Mineralogy

## Abstract

Earth surface redox conditions are intimately linked to the co-evolution of the geosphere and biosphere. Minerals provide a record of Earth’s evolving surface and interior chemistry in geologic time due to many different processes (e.g. tectonic, volcanic, sedimentary, oxidative, etc.). Here, we show how the bipartite network of minerals and their shared constituent elements expanded and evolved over geologic time. To further investigate network expansion over time, we derive and apply a novel metric (weighted mineral element electronegativity coefficient of variation; wMEE_CV_) to quantify intra-mineral electronegativity variation with respect to redox. We find that element electronegativity and hard soft acid base (HSAB) properties are central factors in mineral redox chemistry under a wide range of conditions. Global shifts in mineral element electronegativity and HSAB associations represented by wMEE_CV_ changes at 1.8 and 0.6 billion years ago align with decreased continental elevation followed by the transition from the intermediate ocean and glaciation eras to post-glaciation, increased atmospheric oxygen in the Phanerozoic, and enhanced continental weathering. Consequently, network analysis of mineral element electronegativity and HSAB properties reveal that orogenic activity, evolving redox state of the mantle, planetary oxygenation, and climatic transitions directly impacted the evolving chemical complexity of Earth’s crust.

## Introduction

A crucial link between the geosphere and biosphere in major planetary evolution events is electricity. Energy gained by electron transfer reactions is the driving force of many key Earth systems mechanisms and the foundation of all life processes^1,2^. Reduction/oxidation potential (also known as redox potential) measures the propensity of a chemical species to gain negatively charged electrons and become reduced. Similarly, electronegativity is the tendency of an atom to attract a shared pair of electrons to itself^3^. The electronegativity of each chemical element is based on its electron configuration and nuclear structure, directly influencing the distribution of electron density, redox potential, and reactivity of chemical species.

Hard soft acid base (HSAB) theory was developed to explain reaction mechanisms, pathways, and stability of compounds formed by acids and bases^4^. “Hard” refers to acids and bases that are small, have high charge states, and are non-polarizable, while “soft” refers to acids and bases that are large, have low charge states, and are highly polarizable. Mineral element associations are initiated by many different processes (tectonic, metamorphic, volcanic, sedimentation, atmospheric and oceanic oxygenation, etc.), which contribute to HSAB and element electronegativity interactions in geologic time. Introduced to unify inorganic and organic reaction chemistry^5^, HSAB theory has been applied in concert with electronegativity to develop predictive models of cation exchange of clay surfaces^6^, analyze partitioning of metals in hydrothermal systems^7^, and evaluate the chemical reactivity of magmatic fluids^8^. These applications represent a wide range of temperatures, pressures, and chemical conditions, demonstrating the broad influence of HSAB properties and electronegativity in geochemical processes.

Geological records, such as stable-isotope fractionation (sulfur, nitrogen, carbon), redox-sensitive metals and their isotopic anomalies, represent changes in Earth surface oxidation in geologic time^9–12^. Planetary redox conditions govern the flow of electrons among aqueous chemical species, thus determining which electron transfer reactions and redox sensitive metals could be utilized in metabolic pathways or as metal cofactors on primitive Earth^13,14^. Biological electron transfer processes across the tree of life are catalyzed by a class of proteins called oxidoreductases (e.g. ferredoxin, nitrogenase), which commonly contain transition metals, known as cofactors, in their active sites^15^. However, traditional forms of geochemical evidence used to reconstruct the specific redox conditions that impacted metal cofactor availability and electron transfer processes are limited throughout much of Earth’s history.

Minerals comprise an abundant source of geochemical evidence for characterizing planetary redox conditions throughout Earth history^16,17^. The elemental composition of a given mineral implicitly records information about the chemical speciation and redox state of critical building block elements at the time of mineral formation^18^. The Mineral Evolution Database [MED; https://rruff.info/evolution/^19^; accessed February 3rd, 2020] contains the chemical composition, known redox chemistry, and oldest/maximum known ages of all “5,763” known mineral species. Previous analyses of this extensive data resource have provided a deeper understanding of mineral co-occurrences and facilitated predictions of mineral species that occur on Earth that have yet to be discovered^17^. Interrogating the MED also provides a distinct opportunity to further understanding of planetary redox evolution.

Network analysis has emerged as a useful tool for investigating large scale mineralogical systems by providing a dynamic visualization platform for higher-dimensional analysis of relationships among hundreds of mineral species^20,21^. The examination of shared mineral localities in a network framework reveals topologies of disequilibrium phase assemblages and pathways of mineral reaction series that are embedded within the network^20^. Mineral chemistry network analysis which links minerals with their constituent elements allows for the visualization and analysis of expanding element associations through time in evolving networks^22,23^. As the range of interactions between different chemical species expanded through time, the extent of potential redox reactions and electron transfer processes expanded as well. Recently, we developed a browser-based platform called dragon^24^ that allows for deep-time exploration of the mineral-chemistry network. Here we leverage this network analysis framework to investigate the oxidation of Earth’s crust as recorded in the electronegativity and HSAB chemistry preserved in the mineral record over 4.7 billion years of history on Earth and beyond. The > 4.7 billion year mineral record includes minerals from both terrestrial and extra-terrestrial sources.

## Results

### The expanding network of mineral chemistry

**Figure 1:**
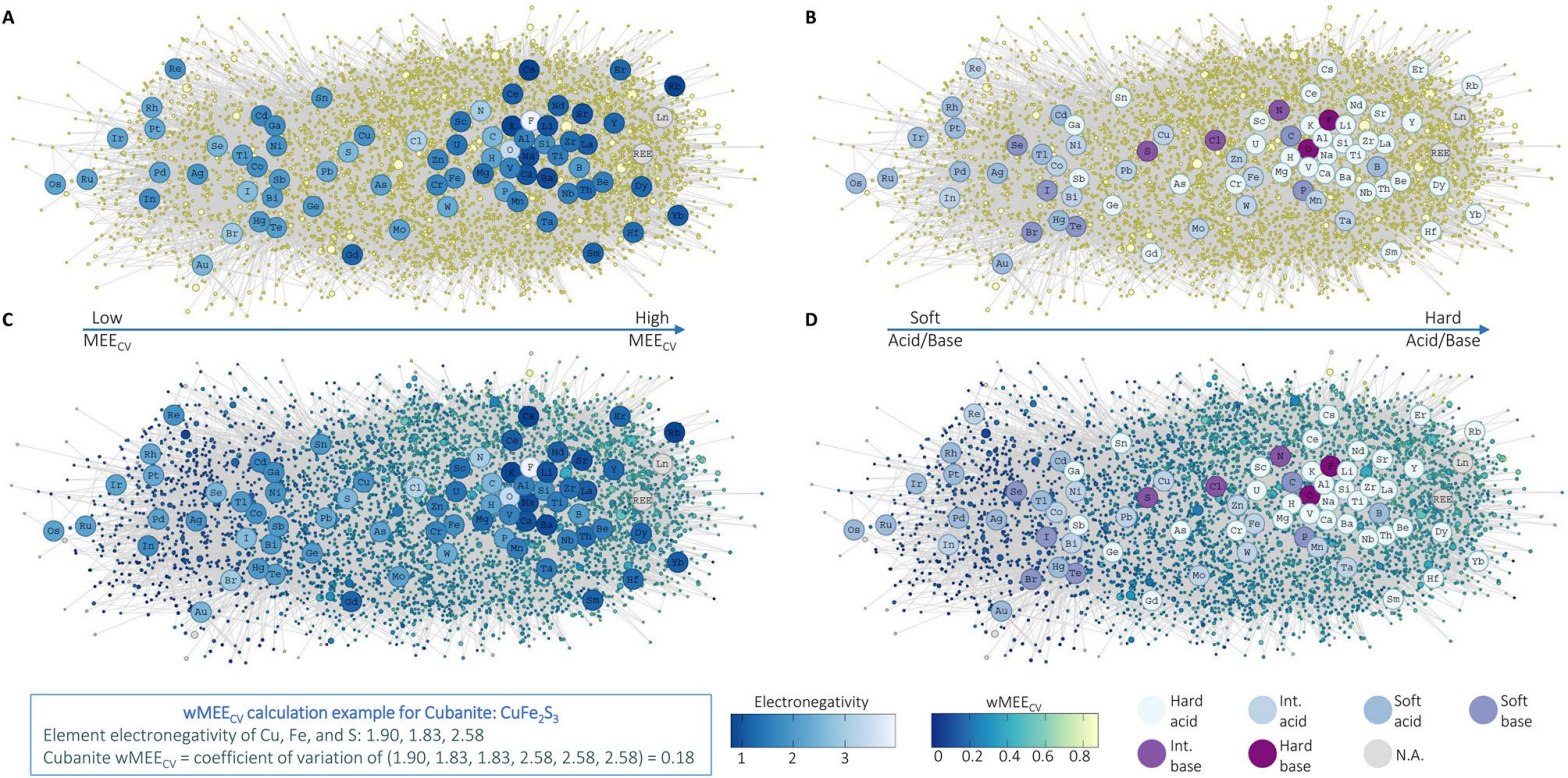
Electronegativity and HSAB dynamics in the full mineral chemistry network. (**A**) Bipartite mineral chemistry network containing all known minerals and their constituent elements. Mineral nodes are represented by small yellow circles, and element nodes are represented by blue scale circles with each element’s chemical symbol. Element nodes are colored by Pauling scale electronegativity^3^. Network lines (“edges”) connect minerals to all of their constituent elements (ex: cubanite—CuFe_2_S_3_ node has network edges connected to Cu, Fe and S). Mineral nodes are sized by the number of known localities. Minerals > 4.33 Ga represent meteorite, asteroid, or pre-solar sources. Node position of the default network layout configuration uses the force-directed Fruchterman-Reingold algorithm^52^. (**B**) The same network as Fig. 1A with element nodes colored by Hard Soft Acid Base theory classification^4^. (**C**) The same network as 1A with minerals colored by weighted Mineral Element Electronegativity Coefficient of Variation (wMEE_CV_). wMEE_CV_ example calculation is shown for the mineral Cubanite (CuFe_2_S_3_). (**D**) The same network as 1B with minerals colored by wMEE_CV_. Arrows indicate transition from low wMEE_CV_ soft acid/base minerals to high wMEE_CV_ hard acid/base minerals. Figure created using dragon version 1.1.0 (https://github.com/sjspielman/dragon).

**Figure 2:**
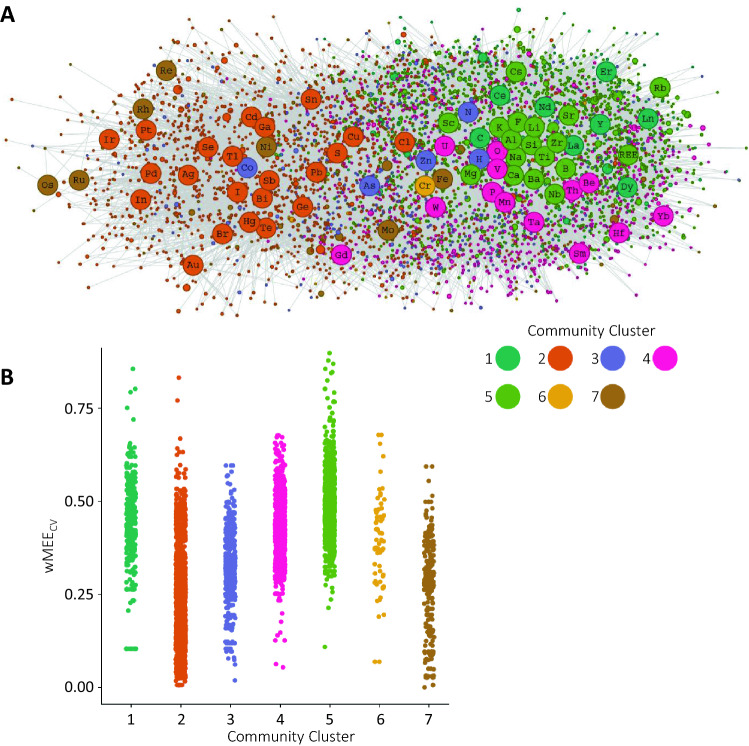
Full mineral chemistry network wMEE_CV_ clustering. (**A**) Bipartite mineral chemistry network containing all known minerals and their constituent elements (same network as Fig. 1) with minerals and elements colored by Louvain community detection cluster^25^. (**B**) A plot of weighted Mineral Element Electronegativity Coefficient of Variation (wMEE_CV_) separated by Louvain community cluster. The wMEE_CV_ values of nearly all network clusters are statistically different from each other by Tukey Test. The only clusters that are not statistically different from each other are clusters 1 and 6, 4 and 6, and 2 and 7 (Supplementary Table S1). Figure created using dragon version 1.1.0 (https://github.com/sjspielman/dragon).

The full mineral chemistry network includes all minerals from 4.7 Ga to present day with known maximum ages, and the constituent elements of the minerals in the network (Fig. 1). The chemistry of the full mineral network largely follows HSAB dynamics, such that hard acid low electronegativity elements (e.g. alkali metals and alkaline Earth metals) commonly form minerals with hard base high electronegativity elements as shown with Louvain community detection (e.g. oxygen and fluorine; Figs. 1, 2). Louvain community detection is ideal for this study because it optimizes modularity when identifying network node communities in the complex mineral chemistry networks^25^. Conversely, many elements with intermediate electronegativities, such as transition metals and larger p-block elements (soft acids and soft bases), form minerals together. Overlap between hard acids/bases with intermediate or soft acids/bases also occurs, but mineral formation generally follows HSAB trends. Minerals with higher wMEE_CV_ values represent hard acid/hard base chemical associations, while minerals with lower wMEE_CV_ values correspond to soft acid/soft base chemistry (Figs. 1B, 2; see methods for wMEE_CV_ calculation). As a result, hard acids and hard bases cluster together, and soft acids and soft bases cluster together in the full mineral network using Louvain community detection (Fig. 2A).

Oxygen containing minerals predominantly have higher wMEE_CV_ values than do non-oxygen containing minerals because oxygen is a hard acid and the second-most electronegative mineral-forming element. The only minerals with higher wMEE_CV_ values than oxygen-containing minerals are fluorine-containing minerals, particularly those that also contain alkali metals and alkaline Earth metals. There are statistically significant differences in the wMEE_CV_ values among the majority of network community clusters, which reflects the links between electronegativity, HSAB dynamics, and mineral redox chemistry (Fig. 2B, Supplementary Table S1). Network clusters 2, 3, and 7 represent soft and intermediate acid and base elements with moderate electronegativity leading to low wMEE_CV_ values. Network clusters 6, 4, 1, and 5 increase in wMEE_CV_ values with increasing electronegativity range and acid/base hardness of mineral forming elements. Clusters 4 and 5 include the largest proportion of minerals that contain hard acids (i.e. alkali metals and alkaline Earth metals), and hard bases (i.e. oxygen and fluorine).

**Figure 3:**
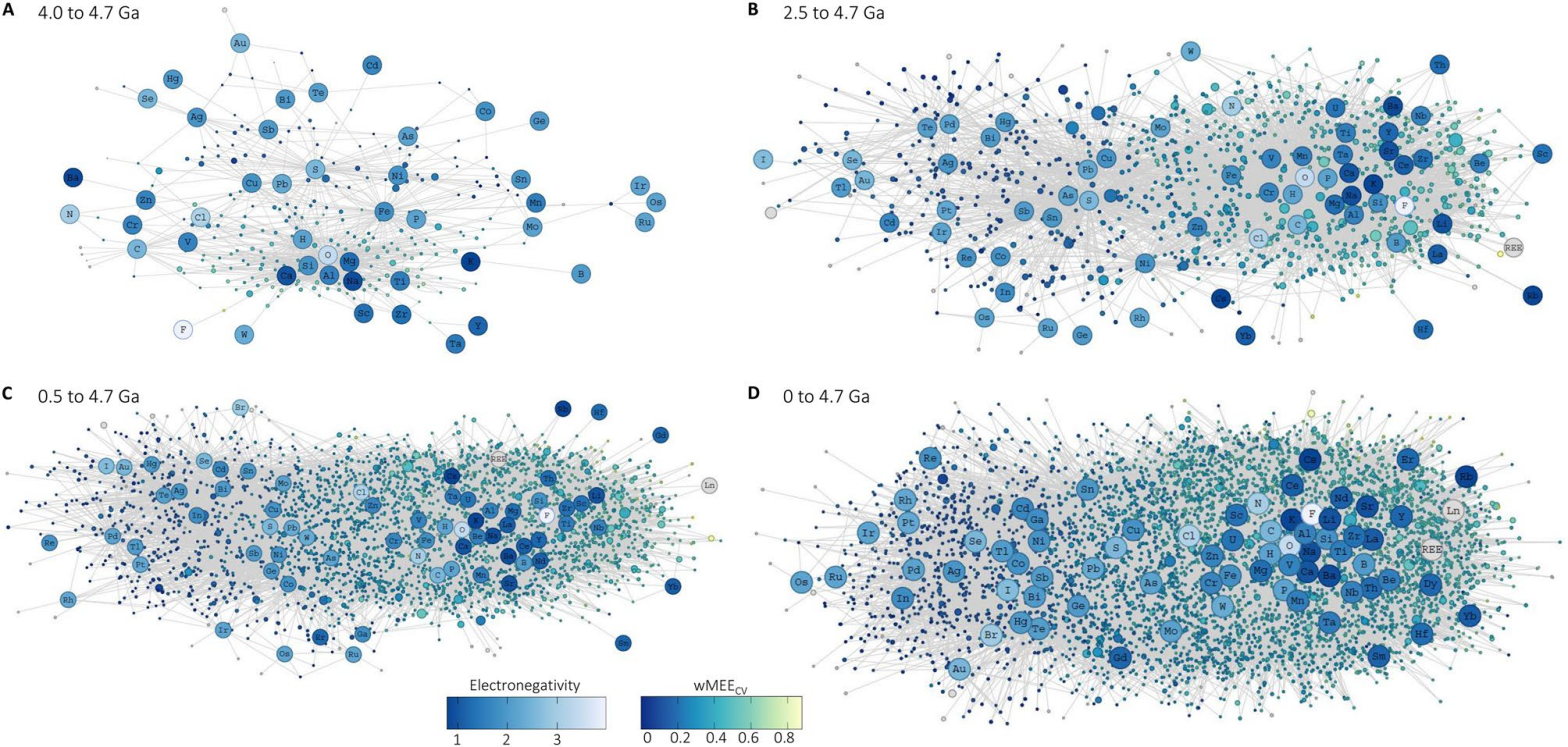
Full mineral chemistry network expansion through geologic time. Bipartite mineral chemistry network containing all minerals and their constituent elements with maximum known ages at (**A**) 4.0–4.7 Ga; (**B**) 2.5–4.7 Ga; (**C**) 0.5–4.7 Ga; (**D**) 0–4.7 Ga. Network lines (“edges”) connect minerals to all of their constituent elements. Element nodes are colored by Pauling scale electronegativity^3^ and minerals nodes are colored by weighted Mineral Element Electronegativity Coefficient of Variation (wMEE_CV_). Mineral nodes are sized by their number of known localities. Figure created using dragon version 1.1.0 (https://github.com/sjspielman/dragon).

The mineral network structure with the segregation of low wMEE_CV_ minerals (soft acid/soft base elements) from high wMEE_CV_ minerals (hard acid/hard base elements) takes shape in the Archean eon and becomes more pronounced through the Proterozoic and Phanerozoic eons (Fig. 3 and Supplementary Figure S1). Through much of Earth history the majority of minerals found at a high number of localities are high wMEE_CV_ minerals, largely due to the increasing presence of oxygen as the predominant anion in mineral species (Fig. 3). Network modularity is highest in the Hadean eon (> 4.0 Ga) when element nodes have fewer network edges with existing minerals, and element nodes do not cluster as closely around oxygen resulting in a more dispersed network (Supplementary Table S2). Network modularity decreases as element and mineral nodes become more connected with each other. Despite the high variability in mineral forming processes associated with the oldest known occurrence of each mineral (i.e. igneous, tectonic, metamorphic, weathering, sedimentary, etc.), element electronegativity and HSAB processes are crucial chemical factors in the full mineral chemistry network structure throughout 4.7 Ga of geologic history.

### Global shifts in mineral electronegativity and HSAB properties

**Figure 4:**
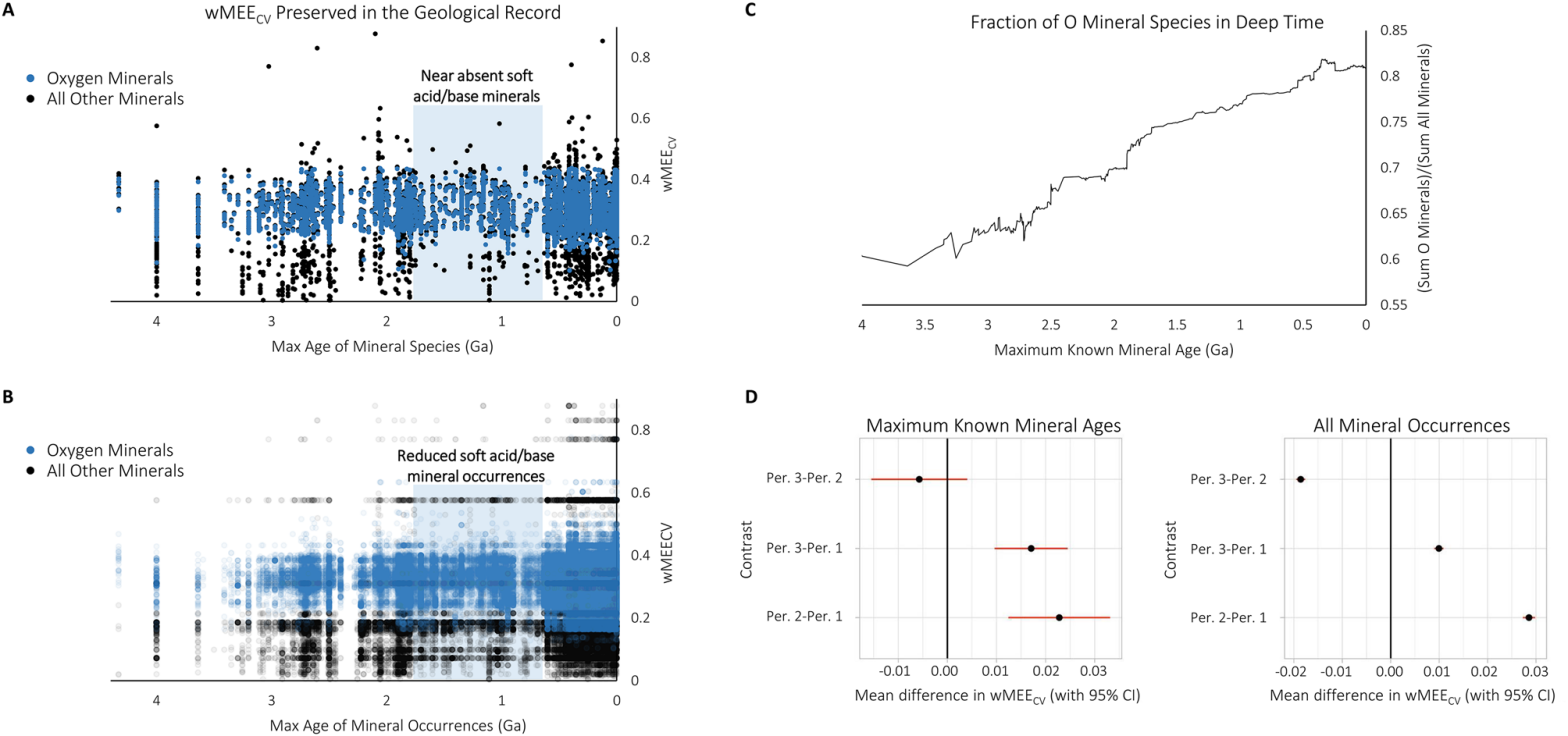
Reduced soft acid/base mineral occurrences from 1.8 to 0.6 Ga. (**A**) Weighted Mineral Element Electronegativity Coefficient of Variation (wMEE_CV_) plotted by maximum known mineral age in billions of years ago (Ga) for 0 to 4.33 Ga. Blue dots represent oxygen containing minerals and black dots represent non-oxygen containing minerals. (**B**) wMEE_CV_ plotted by the maximum age of > 209,000 mineral occurrences in the Mineral Evolution Database in billions of years ago (Ga). Blue dots represent oxygen containing mineral occurrences and black dots represent non-oxygen containing mineral occurrences. (**C**) The ratio of the number of oxygen-containing mineral species to all mineral species through time based on the maximum known ages (Ga) of all minerals. (**D**) Line range plots showing results from ANOVA and post-hoc Turkey test to compare wMEE_cv_ values among the three time periods: period 1 (4.34 Ga < t < 1.8 Ga), period 2 (1.8 Ga < t < 0.6 Ga), and period 3 (0.6 Ga < t < 0 Ga).

The range of wMEE_CV_ values has expanded through time for the maximum known ages of all mineral species (Fig. 4A), and also when the maximum and minimum known ages of > 209,000 mineral occurrences in the MED are considered (Fig. 4B; Supplementary Figure S2). A clear separation is evident between the wMEE_CV_ values of oxygen-containing minerals and non—oxygen-containing minerals resulting from the influence of oxygen’s high electronegativity on wMEE_CV_ values (Fig. 4). A substantial expansion of the number of new oxide and hydroxide mineral species relative to non-oxygen containing minerals following the Whiff of Oxygen and Great Oxidation Event, in addition to oxygen containing minerals formed due to interactions between water and mantle/crustal material, exemplifies the mineral evolution era of bio-mediated mineralogy (< 2.5 Ga)^9–11,18,26^.

The frequency of new mineral species formation and preservation is reduced during the period of 1.8–0.6 Ga, particularly for non-oxygen containing minerals with low wMEE_CV_ values (Fig. 4A). The frequency of total mineral occurrences, especially for non-oxygen containing minerals with low wMEE_CV_ values, is also reduced during the period of 1.8–0.6 Ga (Fig. 4B). Minerals with low wMEE_CV_ values contain soft acids and bases, such as heavy transition metals, heavy p-block metals, and heavy p-block bases (e.g. As, Se, Sb, Te). Low wMEE_CV_ minerals occur throughout the geologic record, but are noticeably absent from 1.8 to 0.6 Ga. The chi-squared tests show that there is a significant association between time and low vs. high wMEE_CV_ abundance (*P* < 2.2e-16 for all occurrences of all minerals; *P* = 7.42e-12 for only the oldest known occurrence of each mineral), further indicating there is an absence of low wMEE_CV_ minerals from 1.8 to 0.6 Ga. (Supplementary Figure S3). Results from ANOVA and post-hoc Tukey tests comparing wMEE_CV_ values over time groups for all mineral occurrences show significant differences (all *P* < 2.2e-16) of wMEE_CV_ among all three time group comparisons, with Group 2 having the highest mean wMEE_CV_. Similarly, results from ANOVA and post-hoc Tukey tests comparing wMEE_CV_ values across time groups for only the oldest known occurrence of each mineral show significant differences between wMEE_CV_ values in Group 1 vs. Group 2 and Group 1 vs. Group 3 (both corrected *P* < 1e-6), but no significant difference between Group 2 vs. Group 3 (Fig. 4D).

The maximum known ages of low wMEE_CV_ soft acid/base minerals occur primarily in mafic, ultramafic, igneous, or volcanic settings prior to 1.8 Ga, representing the redox state of mantle sources. Conversely, after 0.6 Ga there is an increased proportion of low wMEE_CV_ soft acid/base mineral formation maximum known ages associated with sedimentary, hydrothermal, and metamorphic settings (Supplementary Table S3). The majority of minerals with low wMEE_CV_ values (i.e. < 0.11) and maximum known ages between 1.8 and 0.6 Ga occur at 1.108 + 0.001 Ga in the Marathon Deposit of the Coldwell Complex (Supplementary Table S4). The Coldwell Complex is the largest alkaline intrusion associated with the Midcontinent Rift System in North America, containing wide range of rock types, mineral formations, and magmatic isotope signatures^27^. All of the non-oxygen containing low wMEE_CV_ minerals of the Coldwell Complex Marathon Deposit at 1.108 + 0.001 Ga contain either palladium (Pd) or rhodium (Rh). Palladium and rhodium are both second row transition metals, adjacent to each other in the periodic table, and both are soft acid HSAB elements with intermediate electronegativity. This mineralization event coincides with the unique conditions of the Rodinia assembly at 1.3 to 0.9 Ga^28^. However, Pd and Rh commonly originate hosted in the mineral pentlandite and progressive oxidation transforms the pentlandite host to other diverse minerals^29^, which could result in more low wMEE_CV_ minerals than originally formed at a given location.

Various studies tracking δ^13^C stasis and chromium isotope fractionation have shown the presence of stable environmental conditions and low atmospheric oxygen levels during much of the Proterozoic from ~ 1.8 to 0.8 Ga^30–33^. This period also coincides with decreased continental elevation and runoff^34,35^, reduced mineralization and mineral preservation of the intermediate ocean (1.9–1.0 Ga), the assembly of the Rodinia supercontinent (1.3–0.9 Ga), and glaciation/post-glacial oxidation (1.0–0.542 Ga) eras^26,28^. From 4.33 to 1.8 Ga, the range of wMEE_CV_ values expands for mineral occurrences, as does the proportion of oxygen-containing mineral species (Fig. 4). The period from 1.8 to 0.6 Ga is marked by the near absence in origination of non–oxygen-containing mineral species, and the reduced occurrence of minerals with low wMEE_CV_ values. The near absence of low wMEE_CV_ minerals from 1.8 to 0.6 Ga is particularly apparent for chalcogenides. Despite overall reduced mineralization and preservation from 1.8 to 0.6 Ga, there is a greater proportion of oxygen-containing mineral species compared to non–oxygen-containing minerals with maximum known ages during this period, and a greater proportion of total oxygen containing mineral occurrences. Following 0.6 Ga the range of wMEE_CV_ values expands for both mineral species with maximum ages during this period and total mineral occurrences, including the expansion of wMEE_CV_ values for oxygen containing minerals, representing the growing presence of oxygen in Earth’s crustal chemistry due to a wide range of geological processes in the Phanerozoic.

Given the dynamic nature of tectonic recycling of continental plates, there is a greater probability that older rocks and minerals will be subducted and lost to the mantle^36,37^, resulting in a preservation and sampling bias towards younger minerals. Harder less soluble minerals are also likely to be preserved to greater extent than their softer more soluble mineral counterparts, and economically significant minerals are more likely to be sampled and observed than other types of minerals^21^. Mineral deposits can also be altered after the formation of the host lithology, resulting in potentially different ages of the mineral and its setting^38^. Despite potential age, preservation, and economic significance biases, periods of expected increased mineralization are apparent during episodes of known continental assembly [i.e. Kenorland 2.8–2.5 Ga, Columbia 2.0–1.8 Ga, Rodinia 1.3–0.9 Ga, Pannotia 0.54–0.5 Ga, and Pangea 0.4–0.3 Ga^17,28,39^; Fig. 4]. Furthermore, the expansion of wMEE_CV_ through time for mineral species and mineral occurrences, and decreased formation and preservation of low wMEE_CV_ minerals from 1.8 to 0.6 Ga are observed for both maximum known ages of mineral occurrences (Fig. 4B) and minimum known ages of mineral occurrences (Supplementary Figure S2).

## Discussion

Element electronegativity and HSAB interactions are crucial factors in the structure of the full mineral chemistry network, as observed in network clustering, network expansion, and expanding wMEE_CV_ through time (Figs. 1, 2, 3, 4). The wMEE_CV_ metric quantifies differences among different mineral element associations by characterizing intra-mineral variation of element electronegativity values that are unique to every mineral species. Mineral element electronegativity and wMEE_CV_ demonstrate the increasing impact of oxygen on the chemistry of Earth’s crust and mantle at different stages of Earth history (Figs. 3, 4). Mineralization and preservation increases in the Meso- and Neoarchean with the development of plate tectonics, which enhanced interactions between water and both the crust and mantle^40^. The near absence of preserved soft acid/soft base low wMEE_CV_ minerals with maximum ages from 1.8 to 0.6 Ga and the reduced occurrences of soft acid/soft base low wMEE_CV_ (< 0.11) minerals from 1.8 to 0.6 Ga is consistent with diminished orogenic crustal thickening and consequently abated continental runoff^34,35,39^. Additionally, minimal mineralogical innovation and preservation occurred in the intermediate ocean (1.9–1.0 Ga), Rodinia supercontinental assembly (1.3–0.9 Ga), and glaciation/post-glacial oxidation (1.0–0.542 Ga) eras^26,28^.

Soft acids and bases are less reactive than are hard acids and bases^4,5^, and the greatest stability of magmas and crustal materials is achieved with increasing HSAB hardness^41^. Therefore, minimal formation of new mineral species and preservation at 1.8–0.6 Ga would impede the interaction and mineralization of less reactive soft acids and bases to a greater extent than hard acids and bases. The presence of S in fluid magma decreases the hardness of the phase, leading to more stable interactions with soft metals, such as Au, Ag and Cu^41^. However, orogenic quiescence from the late Paleoproterozoic until the mid-Neoproterozoic^35^ would be expected to result in reduced magmatic soft acid–base interactions in the mantle and diminished incorporation in crustal material. Enhanced erosion of Rodinian volcanic arcs and orogens from 1.3 to 0.9 Ga would have primarily resulted in loss of near-surface minerals, which are more oxidized and diverse, and occur in a wider range of environments (e.g. epithermal ore deposits, evaporites, and volcanics) than are sub-surface minerals because of the simpler make-up of the mantle^28^. Despite enhanced surface erosion of oxidized minerals and other diverse surface minerals from 1.3 to 0.9 Ga, there is an increasing fraction of preserved oxygen-containing minerals compared to low wMEE_CV_ minerals during this period and extending through 1.8–0.6 Ga (Fig. 4). Mineralization and wMEE_CV_ values expand in the Phanerozoic with increased atmospheric oxygen concentrations, oxidative weathering, continental weathering, and the colonization of land by plants and animals^42–44^. Living systems are composed mainly of hard elements and hard-hard HSAB interactions, while soft elements are more likely to be biologically toxic^45^, suggesting a stronger biological influence over harder oxygen containing materials as eukaryotic phtosynthesizers proliferated in the Phanerozoic^46,47^.

Element electronegativity and HSAB dynamics are known to be important determining factors in a wide range of geochemical processes including partitioning of trace elements during magma crystallization^48^, cation exchange of clay surfaces^6^, Gibbs free energy of formation for hydrated clay minerals^49^, degradation temperature of agardites (member of mixite mineral group)^50^, partitioning of metals in hydrothermal systems^7^, and chemical reactivity in magmatic fluid intrusions^8^. These mechanisms encompass a wide range of physical and chemical conditions, just as the oldest known occurrences of each mineral in the full mineral chemistry network originated due to a wide range of processes (igneous, tectonic, metamorphic, weathering, sedimentation, etc.). Each mineral species represents unique combinations of electronegativity and HSAB interactions at different points in time, despite contrasting crustal concentrations of each element^51^. Consequently, network analysis of mineral element electronegativity and HSAB properties reveal that orogenic activity, evolving redox state of the mantle, planetary oxidation, and climatic transitions are directly linked with the electronegativity, HSAB properties and redox evolution of Earth’s crust.

## Summary

The chemical composition and alteration of Earth’s crust has been influenced by a wide range of geological and biological processes throughout the planet’s history. Identifying the contributions and interactions of such factors is a major challenge in the geosciences. Network analysis on all known mineral species, their constituent elements, electronegativity, and HSAB interactions throughout Earth’s history illustrates how the network of mineral chemistry expanded and evolved over geologic time, and reveals global shifts in mineral electronegativity 1.8 and 0.6 billion years ago. The observed changes in mineral chemistry are associated with decreased continental elevation, climate transitions, and eventual increase in atmospheric oxygen. Major shifts in mineral element electronegativity and HSAB properties through time reflect evolving planetary redox conditions and Earth system transitions that provide a new guide for interpreting the mineralogy of other planetary bodies as humans explore the solar system.

## Methods

We constructed bipartite networks consisting of two node types, minerals and their constituent elements (Fig. 1), using the R package dragon^24^. Data used in network analysis was obtained from the Mineral Evolution Database (https://rruff.info/evolution/; accessed February 3rd, 2020). The Mineral Evolution Database (MED) contains the nominal chemical formulas, known redox chemistry of mineral constituent elements, and oldest/maximum known ages of all known mineral species. For the majority of mineral localities the tectonic environment is not documented. However, the mineral localities with documented tectonic environment information primarily occur in igneous and mafic–ultramafic settings in the Hadean and Archean, with increasing occurrence of metamorphic and sedimentary settings in the Proterozoic and Phanerozoic. This trend was also observed for low wMEE_CV_ soft acid/base minerals (Table S3). Mineral chemistry bipartite networks consist of mineral nodes and element nodes in which mineral nodes have network connections to all of the constituent elements of that mineral (network lines are referred to as “edges”). For example, the mineral cubanite (CuFe_2_S_3_) node has network edges connected to Cu, Fe and S (Fig. 1). Node position of the default network layout configuration uses the force-directed Fruchterman-Reingold algorithm^52^, which positions nodes based on the number of shared edges throughout the network. Mineral chemistry networks were constructed at different periods in deep time to investigate network expansion, including the time periods 4.0–4.7 Ga (billion years ago), 2.5–4.7 Ga, 0.5–4.7 Ga, and finally present day to 4.7 Ga.

Mineral nodes are sized by the number of known localities to account for different crustal abundances of different mineral species and distinguish between minerals that are major vs. minor components of Earth’s crust. For each network, we performed Louvain community detection analysis^25^ to identify associations between minerals and elements in the full mineral chemistry network (Fig. 2). We calculated weighted Mineral Element Electronegativity coefficient of variation (wMEE_CV_) values and weighted Mineral Element Electronegativity mean (wMEE_μ_) values for a given mineral from the Pauling electronegativity values of the minerals’ constituent elements, weighted by the number of elements in the mineral. Pauling electronegativity was chosen because the electronegativity values are determined using multiple different covalent bonds for a given element^3^. To derive these quantities, therefore, we counted the total number of each element in each mineral based on the IMA mineral formula. For example, the Pauling Scale electronegativity values for the constituent elements of the mineral cubanite (CuFe_2_S_3_) are: Cu = 1.90; Fe = 1.83; S = 2.58. Cubanite contains one Cu atom, two Fe atoms, and three S atoms, totaling six atoms. Therefore, we calculate the wMEE_CV_ from the six values 1.9, 1.83, 1.83, 2.58, 2.58, and 2.58. Specifically, to calculate wMEE_μ_, we perform: (1*1.9 + 2*1.83 + 3*2.58)/6 = 2.21. To calculate wMEE_CV_, we calculate the standard deviation of those six values (0.4), and divide by their mean (2.21), to obtain a final wMEE_CV_ = 0.18. The calculated wMEE_CV_ and wMEE_μ_ values are available in the Supplementary Dataset. We excluded from calculations any mineral for which the IMA formula differed from the RRUFF formula to ensure consistent redox information, as well as any minerals with ambiguous numbers of elements [e.g., henryite with a formula (Cu,Ag)_3+x_Te_2_ (x ~ 0.4) would be excluded]. For any minerals with a defined range of number of elements, we assumed the average; for example, we assume that bauranoite (BaU_2_O_7_·4-5H_2_O) is complexed with 4.5 waters.

For any mineral which can interchangeably contain different elements, we assumed an equal proportion of those options. For example, the mineral urvantsevite, Pd(Bi,Pb)_2_, can interchangeably contain two of either bismuth (Bi) or lead (Pb) atoms. To tabulate the total number of elements, we consider this formula as equivalent to: Pd(Bi_0.5_Pb_0.5_)_2_. After excluding all ambiguous mineral formulas, we were able to calculate the total numbers of each constituent element for 4579 minerals in the MED. Using the tabulated relative proportions of elements, we calculated both the weighted mean (wMEE_μ_) and weighted coefficient of variation (wMEE_CV_) for each of those 4579 minerals. The calculated wMEE_CV_ and wMEE_μ_ values are part of dragon version > 1.1.0, and the code for calculations of the wMEE_CV_ and wMEEμ values is archived in https://github.com/sjspielman/dragon. The calculation of wMEE_CV_ values includes chalcogenide and sulfosalt groups that do not necessarily include strict electron donor and electron acceptor elements in the mineral structure, such as pyrite (FeS_2_) which contains is a combination of Fe–S and S–S bonds^53^.

Statistical analysis of mineral wMEE_CV_ values was performed using R^54^. We defined three age groups as Group 1 (4.34 Ga < t < 1.8 Ga), Group 2 (1.8 Ga < t_2_ < 0.6 Ga) and Group 3 (0.6 Ga < t_3_ < 0 Ga) to analyze mineral wMEE_CV_ values over time. We further define “low wMEE_CV_” as wMEE_CV_ values < 0.11, and “high wMEE_CV_” as wMEE_CV_ values >  = 0.11. We selected this boundary because minerals with wMEE_CV_ values below 0.11 do not contain oxygen, and chalcogenides minerals with wMEE_CV_ values below 0.11 almost exclusively do not contain first row transition metals.

We compared the wMEE_CV_ distributions across age groups using two approaches: (a) ANOVA with a post-hoc Tukey test to ascertain whether wMEE_CV_ values differ among time periods, and (b) chi-squared contingency table analyses to ascertain whether there is an association between time group and low/high wMEE_CV_ categories. Analyses were performed on two versions of mineral data. First, we considered only a single occurrence of each mineral based on its oldest known age. Second, we considered all occurrences of all minerals regardless of their oldest known age. Code to reproduce statistical analyses and associated figures is freely available from the GitHub repository https://github.com/sjspielman/wmeecv_hsab_analysis.

When considering HSAB dynamics, it should be noted that hardness of a given element is different at the different redox states. For example, S^2−^ is a soft highly polarizable ion, and S^6+^ is a hard ion with low polarizability. Therefore, electronegativity does not take into account the hardness of different ions. However, minerals that contain S^6+^ ion(s) also contain O^2−^ interacting directly with S^6+^. Since O^2−^ is a hard base, the presence of O in the mineral formula adds to the overall hardness of the mineral due in part to the presence of S^6+^ in the mineral. Minerals that contain S^2−^ are much more likely to contain soft/intermediate acids, which adds to the overall softness of the mineral. Therefore, by including the weighted electronegativities of all elements in the mineral, wMEE_CV_ does account for the relative hardness or softness of different ions of the same element.

## Supplementary Information

Supplementary Information 1.

Supplementary Information 2.

Supplementary Information 3.

Supplementary Information 4.

Supplementary Information 5.

## Data Availability

All code and associated data used for initial wMEE_CV_ calculations is available within the dragon package (https://github.com/sjspielman/dragon), and further additional code to perform statistical analyses of wMEE_CV_ are available here https://github.com/sjspielman/wmeecv_hsab_analysis. MED data used by and cached within dragon is publicly available from https://rruff.info/evolution/.
