# Supplementary figures and images for: The expanding network of mineral chemistry throughout earth history reveals global shifts in crustal chemistry during the Proterozoic
Source: Sci Rep. 2022 Mar 23;12:4956. doi: 10.1038/s41598-022-08650-x (PMC8943050; doi:10.1038/s41598-022-08650-x)

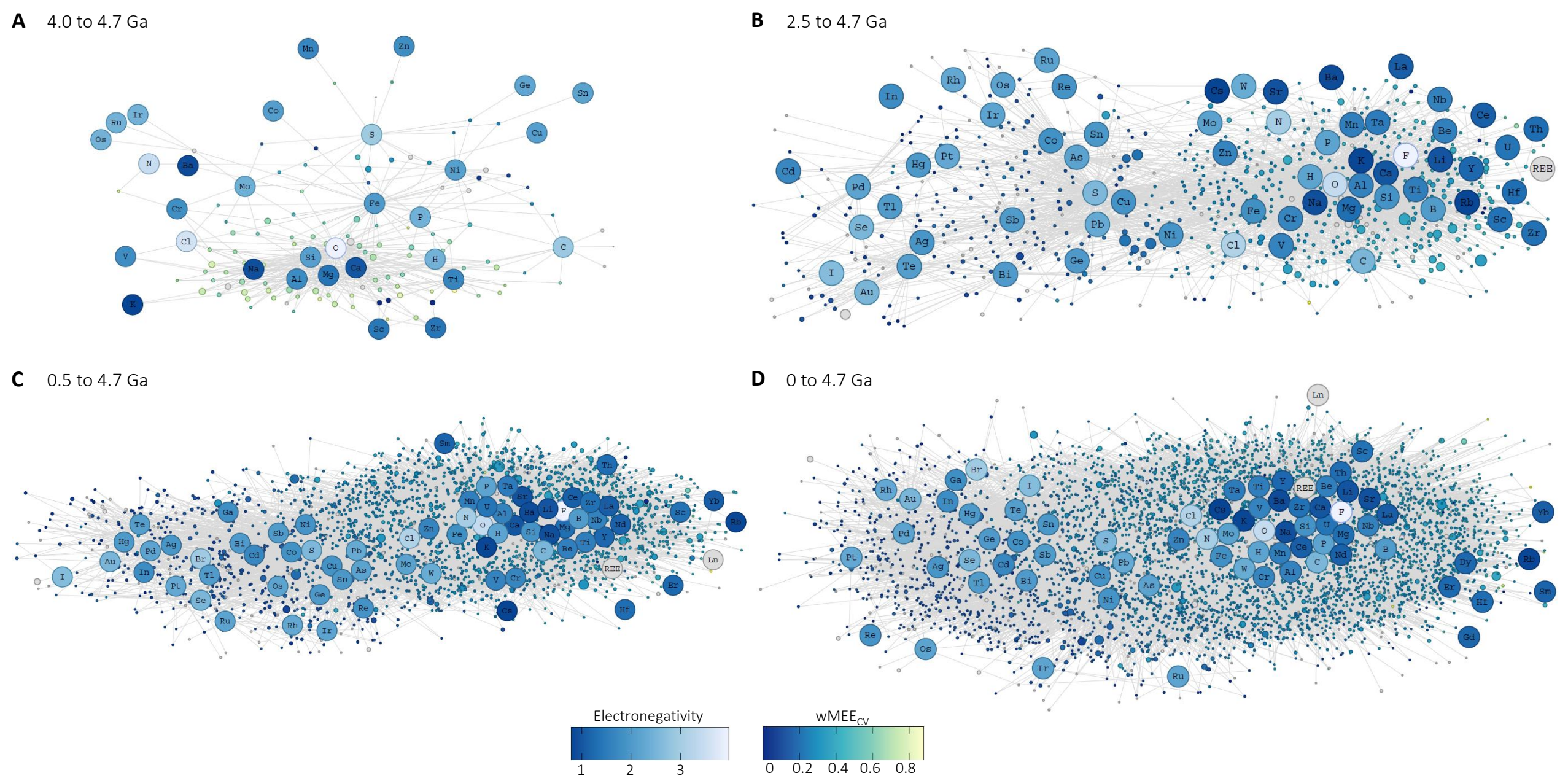

Supplement: Supplementary file 1 — Supplementary Information 1. [file 41598_2022_8650_MOESM1_ESM.pdf]

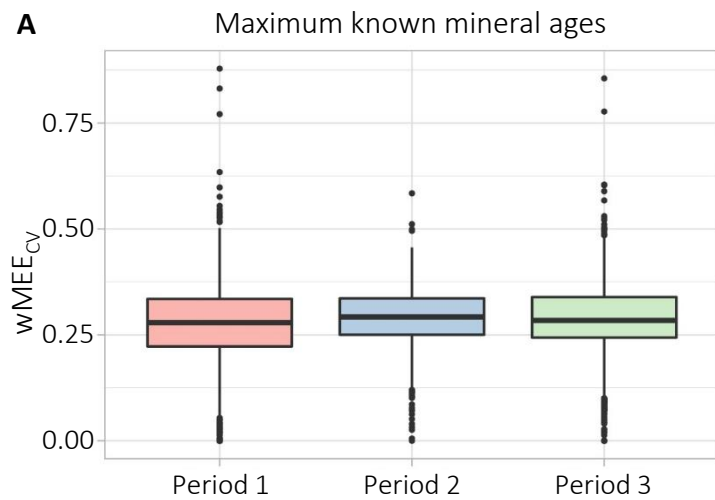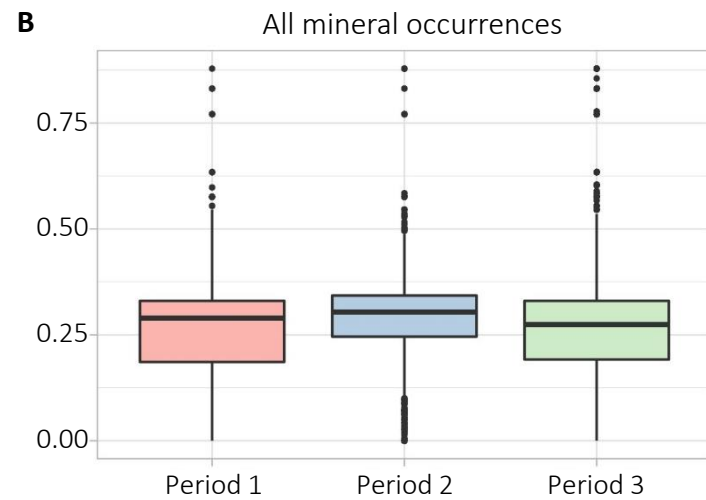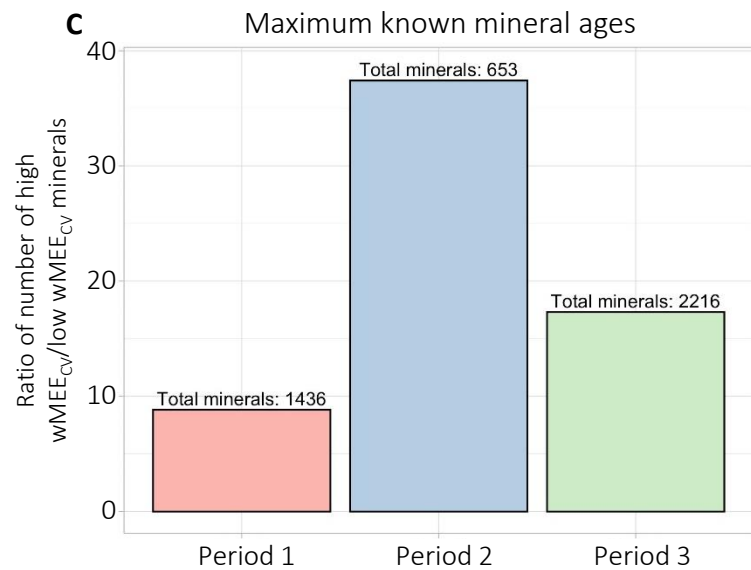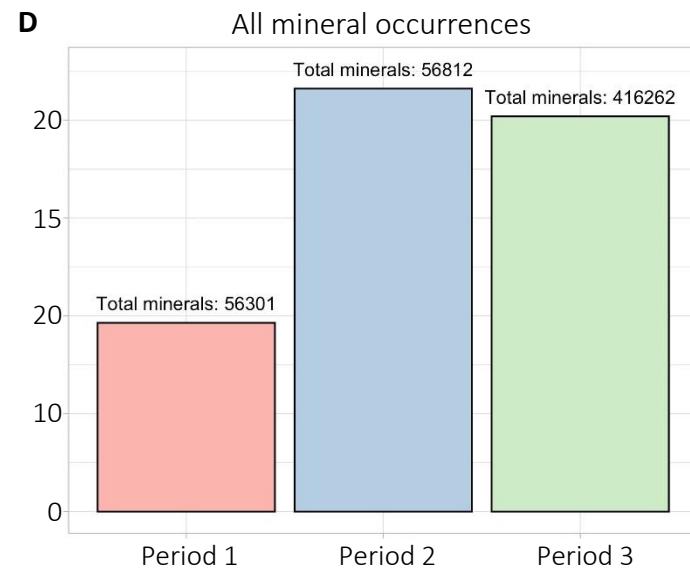

Supplement: Supplementary file 3 — Supplementary Information 3. [file 41598_2022_8650_MOESM3_ESM.pdf]
