# Supplementary material for: The expanding network of mineral chemistry throughout earth history reveals global shifts in crustal chemistry during the Proterozoic
Source: Sci Rep. 2022 Mar 23;12:4956. doi: 10.1038/s41598-022-08650-x (PMC8943050; doi:10.1038/s41598-022-08650-x)
Supplement: Supplementary file 2 — Supplementary Information 2. [file 41598_2022_8650_MOESM2_ESM.pdf]

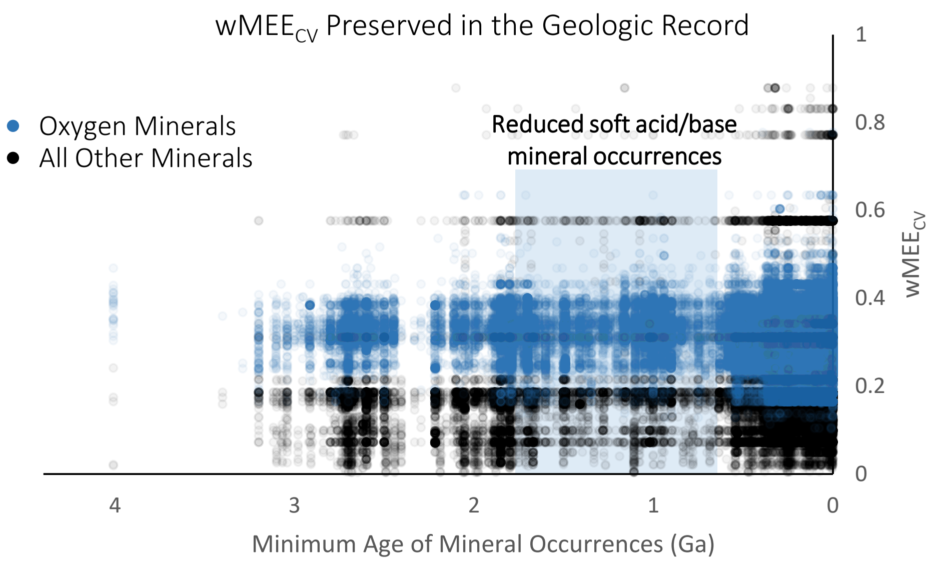
wMEE$_{CV}$ Preserved in the Geologic Record
Oxygen Minerals
All Other Minerals
Reduced soft acid/base mineral occurrences
1
0.8
0.6
0.4
0.2
0
wMEE$_{CV}$
4
3
2
1
0
Minimum Age of Mineral Occurrences (Ga)
