# Supplementary material for: The expanding network of mineral chemistry throughout earth history reveals global shifts in crustal chemistry during the Proterozoic
Source: Sci Rep. 2022 Mar 23;12:4956. doi: 10.1038/s41598-022-08650-x (PMC8943050; doi:10.1038/s41598-022-08650-x)
Supplement: Supplementary file 5 — Supplementary Information 5. [file 41598_2022_8650_MOESM5_ESM.docx]

**The expanding network of mineral chemistry throughout Earth history reveals global shifts in crustal chemistry during the Proterozoic**

**Supplementary Material**

*Eli K. Moore^1^, Josh J. Golden^2^, Shaunna M. Morrison^3^, Jihua Hao^4,5,6^, Stephanie J. Spielman^7^

^1^Department of Environmental Science, School of Earth and the Environment, Rowan University, Glassboro, NJ, United States

^2^Department of Geosciences, University of Arizona, Tucson, AZ Arizona, United States

^3^Earth and Planets Laboratory, Carnegie Institution for Science, Washington, DC, United States

^4^CAS Key Laboratory of Crust-Mantle Materials and Environments, School of Earth and Space Sciences, University of Science and Technology of China, Hefei 230026, China

^5^CAS Center for Excellence in Comparative Planetology, USTC, Hefei 230026, Anhui, China

^6^Department of Marine and Coastal Sciences, Rutgers University, New Brunswick, NJ, United States

^7^Department of Biological Sciences, College of Science and Mathematics, Rowan University, Glassboro, NJ, United States

*Corresponding Author: [mooreek@rowan.edu](mailto:mooreek@rowan.edu)

**SUPPLEMENTARY MATERIAL**

**wMEE_CV_ Calculation**

We provide an example weighted Mineral Element Electronegativity Coefficient of Variation (wMEE_CV_) and weighted Mineral Element Electronegativity Mean (wMEE_μ_) calculation with the mineral cubanite, whose IMA chemical formula is CuFe_2_S_3_. The Pauling Scale electronegativity values for Cu, Fe, and S are: Cu = 1.90; Fe = 1.83; S = 2.58. Cubanite contains one Cu atom, two Fe atoms, and three S atoms, totaling 6 atoms. Therefore, we calculate the wMEE_CV_ from the six values 1.9, 1.83, 1.83, 2.58, 2.58, and 2.58. Specifically, to calculate wMEE_μ_, we perform: (1*1.9 + 2*1.83 + 3*2.58)/6 = 2.21. To calculate wMEE_CV_, we calculate the standard deviation of those six values (0.4), and divide by their mean (2.21), to obtain a final wMEE_CV_ = 0.18. The calculated wMEE_CV_ and wMEE_μ_ values are available in Supplementary Spreadsheet S1.

**Table S1.** Tukey Test results of wMEE_CV_ values for network Louvain community detection analysis clusters in Figure 2.

| Cluster comparison | Estimated effect size difference | 95% CI lower bound | 95% CI upper bound | P-value |
| --- | --- | --- | --- | --- |
| 2-1 | -0.116167 | -0.13025 | -0.102084 | <0.001 |
| 3-1 | -0.059299 | -0.075741 | -0.042858 | <0.001 |
| 7-1 | -0.10385 | -0.123387 | -0.084312 | <0.001 |
| 3-2 | 0.056868 | 0.044305 | 0.069431 | <0.001 |
| 4-2 | 0.092159 | 0.082038 | 0.10228 | <0.001 |
| 5-2 | 0.136354 | 0.12721 | 0.145498 | <0.001 |
| 6-2 | 0.094577 | 0.065219 | 0.123935 | <0.001 |
| 4-3 | 0.035291 | 0.022084 | 0.048498 | <0.001 |
| 5-3 | 0.079486 | 0.067012 | 0.09196 | <0.001 |
| 7-3 | -0.04455 | -0.063022 | -0.026078 | <0.001 |
| 5-4 | 0.044194 | 0.034184 | 0.054204 | <0.001 |
| 7-4 | -0.079842 | -0.096747 | -0.062936 | <0.001 |
| 7-5 | -0.124036 | -0.140376 | -0.107696 | <0.001 |
| 7-6 | -0.082259 | -0.11459 | -0.049929 | <0.001 |
| 4-1 | -0.024008 | -0.038668 | -0.009348 | <0.001 |
| 5-1 | 0.020186 | 0.006183 | 0.03419 | <0.001 |
| 6-5 | -0.041776 | -0.071096 | -0.012456 | <0.001 |
| 6-3 | 0.037709 | 0.00715 | 0.068269 | 0.005 |
| 7-2 | 0.012318 | -0.00409 | 0.028726 | 0.288 |
| 6-1 | -0.02159 | -0.052805 | 0.009625 | 0.389 |
| 6-4 | 0.002418 | -0.027221 | 0.032057 | 0.999 |

**Table S2.** Network metrics of the expanding full mineral chemistry network in Figures 1-3.

| Time period (Ga) | Network modularity | # Element nodes | # Mineral nodes | # Edges | # Edges/ Element | # Edges/ Mineral |
| --- | --- | --- | --- | --- | --- | --- |
| 4.0 to 4.7 | 0.391 | 46 | 232 | 795 | 17.3 | 3.4 |
| 3.5 to 4.7 | 0.391 | 48 | 275 | 934 | 19.5 | 3.4 |
| 3.0 to 4.7 | 0.381 | 57 | 412 | 1473 | 25.8 | 3.6 |
| 2.5 to 4.7 | 0.355 | 66 | 980 | 3953 | 59.9 | 4.0 |
| 2.0 to 4.7 | 0.343 | 68 | 1299 | 5354 | 78.7 | 4.1 |
| 1.5 to 4.7 | 0.306 | 71 | 1885 | 8153 | 114.8 | 4.3 |
| 1.0 to 4.7 | 0.301 | 72 | 2194 | 9753 | 135.5 | 4.4 |
| 0.5 to 4.7 | 0.289 | 73 | 2723 | 12381 | 169.6 | 4.5 |
| 0 to 4.7 | 0.276 | 74 | 4786 | 22797 | 308.1 | 4.8 |

**Table S3.** Chemical formula, maximum ages in billions of years old (Ga), and paragenetic mode of low wMEE_CV_ soft acid/base minerals.

| Mineral | Chemical Formula | Maximum Age (Ga) | Paragenetic Mode |
| --- | --- | --- | --- |
| Hexamolybdenum | (Mo,Ru,Fe,Ir,Os) | 4.7 | meteorite |
| Awaruite | Ni_3_Fe | 4.62 | meteorite |
| Taenite | (Fe,Ni) | 4.62 | meteorite |
| Tetrataenite | FeNi | 4.62 | meteorite |
| Barringerite | Fe_2_P | 4.62 | meteorite |
| Nickelphosphide | Ni_3_P | 4.62 | meteorite |
| Wairauite | CoFe | 4.5685 | meteorite |
| Monipite | MoNiP | 4.56761 | meteorite |
| Tellurobismuthite | Bi^3+^_2_Te^2-^_3_ | 4 | volcanogenic massive sulfide deposit |
| Breithauptite | NiSb | 4 | serpentinised peridotite |
| Hessite | Ag_2_Te | 4 | volcanogenic massive sulfide deposit |
| Mattagamite | CoTe_2_ | 4 | volcanogenic massive sulfide deposit |
| Altaite | Pb^2+^Te^2-^ | 4 | volcanogenic massive sulfide deposit |
| Frohbergite | FeTe_2_ | 4 | ore deposit |
| Skutterudite | Co^3+^As_3_ | 4 | volcanogenic massive sulfide deposit |
| Sperrylite | PtAs_2_ | 3.64 | ophiolite |
| Kotulskite | Pd(Te,Bi)_2-x_ (x â‰ˆ 0.4) | 3.64 | ophiolite |
| Sobolevskite | PdBi | 3.64 | ophiolite |
| Nickelskutterudite | (Ni^4+^,Co^3+^,Fe^3+^)As_3_ | 3.64 | ophiolite |
| Maucherite | Ni_11_As_8_ | 3.64 | ophiolite |
| Nickeline | NiAs | 3.64 | ophiolite |
| Orcelite | Ni_4.77_As_2_ | 3.64 | ophiolite |
| Safflorite | (Co^2+^,Fe^2+^,Ni^2+^)As_2_ | 3.64 | ophiolite |
| Merenskyite | PdTe_2_ | 3.254 | mafic |
| Coloradoite | Hg^2+^Te^2-^ | 3.254 | mafic |
| Mertieite-II | Pd_8_Sb_2.5_As_0.5_ | 3.254 | mafic |
| Sudburyite | PdSb | 3.254 | mafic |
| Moncheite | Pt(Te,Bi)_2_ | 3.254 | mafic |
| Potarite | PdHg | 3.254 | mafic |
| Geversite | PtSb_2_ | 3.254 | mafic |
| Braggite | PtS | 3.254 | mafic |
| Hollingworthite | Rh^3+^(AsS)^3-^ | 3.254 | mafic |
| Michenerite | PdBiTe | 3.1 | ultramafic |
| Stibiopalladinite | Pd_5_Sb_2_ | 3.1 | ultramafic |
| Melonite | NiTe_2_ | 3.1 | ultramafic |
| Irarsite | Ir^3+^(AsS)^3-^ | 3.1 | ultramafic |
| Iridarsenite | IrAs_2_ | 3.074 | metamorphic |
| Dyscrasite | Ag_3.2_Sb_0.8_ | 3.074 | metamorphic |
| Ruthenarsenite | (Ru,Ni)As | 3.074 | metamorphic |
| Cooperite | PtS | 3.074 | metamorphic |
| Osarsite | Os^3+^(AsS)^3-^ | 3.074 | metamorphic |
| Telluropalladinite | Pd_9_Te_4_ | 2.941 | volcano-sedimentary |
| Temagamite | Pd_3_HgTe_3_ | 2.941 | volcano-sedimentary |
| Atheneite | Pd_2_(As_0.75_Hg_0.25_) | 2.941 | volcano-sedimentary |
| Platarsite | Pt^3+^(AsS)^3-^ | 2.941 | volcano-sedimentary |
| Arsenopalladinite | Pd_8_As_3_ | 2.906 | mafic-ultramafic |
| Palladoarsenide | Pd_2_As | 2.906 | mafic-ultramafic |
| Stillwaterite | Pd_8_As_3_ | 2.906 | mafic-ultramafic |
| Keithconnite | Pd_20_Te_7_ | 2.906 | mafic-ultramafic |
| Kojonenite | Pd_7-x_SnTe_2_ (0.3 â‰¤ x â‰¤ 0.8) | 2.906 | mafic-ultramafic |
| Palarstanide | Pd_5_(Sn,As)_2_ | 2.906 | mafic-ultramafic |
| Menshikovite | Pd_3_Ni_2_As_3_ | 2.906 | mafic-ultramafic |
| Atokite | Pd_3_Sn | 2.906 | mafic-ultramafic |
| Stannopalladinite | Pd_3_Sn_2_ | 2.906 | mafic-ultramafic |
| Hedleyite | Bi_7_Te_3_ | 2.858 | metamorphic-metasomatic |
| Tsumoite | BiTe | 2.858 | metamorphic-metasomatic |
| Volynskite | AgBiTe_2_ | 2.858 | metamorphic-metasomatic |
| Rucklidgeite | Pb^2+^Bi^3+^_2_Te^2-^_4_ | 2.858 | metamorphic-metasomatic |
| Stibarsen | SbAs | 2.816 | metamorphic |
| Nisbite | NiSb_2_ | 2.8 | oceanic arc, subduction |
| Rutheniridosmine | (Ir,Os,Ru) | 2.7499 | mafic-felsic |
| Stutzite | Ag5-xTe3 (x = 0.24-0.36) | 2.739 | volcanic |
| Allargentum | Ag_1-x_Sb_x_ (x=0.09-0.16) | 2.73 | volcanogenic massive sulfide deposit |
| Tellurantimony | Sb^3+^_2_Te^2-^_3_ | 2.725 | volcanogenic massive sulfide deposit |
| Pilsenite | Bi_4_Te_3_ | 2.724 | volcanic (basaltic and dacitic) |
| Tungstenite | WS_2_ | 2.716 | volcanogenic massive sulfide deposit |
| Rickardite | Cu_2.64-3.00_Te_2_ | 2.71 | mafic-ultramafic |
| Oregonite | FeNi_2_As_2_ | 2.707 | mafic-ultramafic |
| Omeiite | OsAs_2_ | 2.7 | mafic-ultramafic |
| Weissite | Cu_2-x_Te | 2.7 | andesitic tuffs, carbonaceous siltstones, and amygdaloidal andesitic lavas |
| Isomertieite | Pd_11_Sb_2_As_2_ | 2.6609 | metavolcanic |
| Sudovikovite | PtSe_2_ | 2.6609 | metavolcanic |
| Palladseite | Pd_17_Se_15_ | 2.6609 | metavolcanic |
| Kalgoorlieite | As_2_Te_3_ | 2.638 | hydrothermal |
| Palladobismutharsenide | Pd_2_(As,Bi) | 2.6083 | igneous |
| Telargpalite | (Pd,Ag)_3_Te | 2.6083 | igneous |
| Rustenburgite | Pt_3_Sn | 2.6083 | igneous |
| Nielsenite | PdCu_3_ | 2.507 | mafic-ultramafic |
| Niggliite | PtSn | 2.507 | mafic-ultramafic |
| Clinosafflorite | Co^2+^As_2_ | 2.501 | meta-volcanosedimentary |
| Anduoite | RuAs_2_ | 2.5 | volcanogenic massive sulfide deposit |
| Moschellandsbergite | Ag_2_Hg_3_ | 2.5 | volcanogenic massive sulfide deposit |
| Vulcanite | CuTe | 2.5 | volcanogenic massive sulfide deposit |
| Vincentite | Pd_3_As | 2.49 | mafic-ultramafic |
| Tornroosite | Pd11As2Te2 | 2.49 | mafic-ultramafic |
| Froodite | PdBi_2_ | 2.49 | mafic-ultramafic |
| Maslovite | PtBiTe | 2.49 | mafic-ultramafic |
| Sopcheite | Ag_4_Pd_3_Te_4_ | 2.49 | mafic-ultramafic |
| Majakite | PdNiAs | 2.49 | mafic-ultramafic |
| Paolovite | Pd_2_Sn | 2.461 | mafic-ultramafic |
| Lukkulaisvaaraite | Pd_14_Ag_2_Te_9_ | 2.4439 | magmatic |
| Taimyrite-I | (Pd,Cu,Pt)_3_Sn | 2.4439 | magmatic |
| Mertieite-I | Pd_5+x_(Sb,As)_2-x_ (x = 0.1-0.2) | 2.254 | mafic-gabbro |
| Rammelsbergite | NiAs_2_ | 2.223 | igneous |
| Pararammelsbergite | NiAs_2_ | 2.2229 | igneous |
| Langisite | CoAs | 2.211 | igneous |
| Henryite | (Cu,Ag)_3+x_Te_2_ (x~0.4) | 2.157 | igneous |
| Empressite | AgTe | 2.06 | mafic-ultramafic |
| Cabriite | Pd_2_CuSn | 2.06 | magmatic |
| Bowieite | Rh^3+^_2_S^2-^_3_ | 2.058 | mafic |
| Miassite | Rh_17_S_15_ | 2.058 | mafic |
| Polarite | Pd(Bi,Pb) | 2.05 | mafic-ultramafic |
| Insizwaite | PtBi_2_ | 2.05 | mafic-ultramafic |
| Stistaite | SnSb | 2.038 | magmatic |
| Borovskite | Pd_3_SbTe_4_ | 2.015 | metamorphic-ultramaphic |
| Luberoite | Pt_5_Se_4_ | 1.983 | igneous |
| Algodonite | Cu_1-x_As_x_ (x â‰ˆ 1/7) | 1.96 | na |
| Kieftite | CoSb_3_ | 1.924 | magmatic |
| Naldrettite | Pd_2_Sb | 1.918 | mafic-ultramafic |
| Ungavaite | Pd_4_Sb_3_ | 1.918 | mafic-ultramafic |
| Kolymite | Cu_7_Hg_6_ | 1.899 | porphyry |
| Domeykite | Cu_3_As | 1.898 | metamoorphic-metavolcanic |
| Koutekite | Cu_5_As_2_ | 1.898 | metamoorphic-metavolcanic |
| Ruarsite | Ru^3+^(AsS)^3-^ | 1.85 | mafic-ultramafic |
| Oenite | CoSbAs | 1.83 | metamorphic |
| Domeykite-beta | Cu3As | 1.825 | volcanic-hydrothermal |
| Paradocrasite | Sb_3_As | 1.804 | granitic pegmatite |
| Seinajokite | FeSb2 | 1.804 | granitic pegmatite |
| Eugenite | Ag_11_Hg_2_ | 1.8 | volcanogenic massive sulfide deposit |
| Luanheite | Ag_3_Hg | 1.8 | volcanogenic massive sulfide deposit |
| Paraschachnerite | Ag_1.2_Hg_0.8_ | 1.8 | metamorphic |
| Schachnerite | Ag_1.1_Hg_0.9_ | 1.8 | metamorphic |
| Suessite | Fe_3_Si | 0.5365 | n.a. |
| Perryite | (Ni,Fe)_8_(Si,P)_3_ | 0.5365 | n.a. |
| Shuangfengite | IrTe_2_ | 0.528 | metamorphic-hydrothermal |
| Mayingite | IrBiTe | 0.528 | metamorphic-hydrothermal |
| Krutovite | NiAs_2_ | 0.444 | volcanogenic massive sulfide deposit |
| Nisnite | Ni_3_Sn | 0.44 | metasomatic |
| Vavrinite | Ni2SbTe2 | 0.428 | magmatic |
| Modderite | CoAs | 0.419 | hydrothermal |
| Ravatite | C_14_H_10_ | 0.408 | sedimentary |
| Leadamalgam | HgPb_2_ | 0.367 | mafic-ultramafic |
| Kutinaite | Ag_6_Cu_14_As_7_ | 0.359 | sedimentary |
| Belendorffite | Cu_7_Hg_6_ | 0.354 | n.a. |
| Zhanghengite | CuZn | 0.34 | mafic-ultramafic |
| Fichtelite | C_19_H_34_ | 0.3376 | magmatic-metamorphic |
| Gupeiite | Fe_3_Si | 0.32 | igneous |
| Naquite | FeSi | 0.32 | igneous |
| Xifengite | Fe_5_Si_3_ | 0.32 | igneous |
| Ferchromide | Cr_1.5_Fe_0.2_ | 0.32 | igneous |
| Urvantsevite | Pd(Bi,Pb)_2_ | 0.296 | continental arc |
| Novakite | (Cu,Ag)21As10 | 0.26 | sedimentary |
| Qusongite | WC | 0.2521 | mafic-ultramafic |
| Chromferide | Fe_1.5_Cr_0.2_ | 0.2521 | mafic-ultramafic |
| Danbaite | CuZn_2_ | 0.2521 | mafic-ultramafic |
| Stumpflite | PtSb | 0.2513 | mafic-ultramafic |
| Pasavaite | Pd3Pb2Te2 | 0.2513 | mafic-ultramafic |
| Norilskite | (Pd,Ag)_7_Pb_4_ | 0.251 | mafic-ultramafic |
| Tatyanaite | (Pt,Pd,Cu)_9_Cu_3_Sn_4_ | 0.2469 | magmatic sulfide deposit |
| Borishanskiite | Pd_1+x_(As,Pb)_2_ (x = 0.0-0.2) | 0.2469 | magmatic sulfide deposit |
| Linzhiite | FeSi_2_ | 0.214 | granitic pegmatite |
| Idrialite | C_22_H_14_ | 0.164 | oceanic arc, subduction |
| Kochkarite | Pb^2+^Bi^3+^_4_Te^2-^_7_ | 0.145 | volcanogenic massive sulfide deposit |
| Luobusaite | Fe_0.84_Si_2_ | 0.1414 | mafic |
| Polkanovite | Rh_12_As_7_ | 0.1414 | mafic |
| Zangboite | TiFeSi_2_ | 0.1414 | mafic |
| Sorosite | Cu_1.1-1.2_(Sn,Sb) | 0.0899 | porphyry |
| Mazzettiite | Ag_3_HgPbSbTe_5_ | 0.02782 | caldera |
| Hartite | C_20_H_34_ | 0.023 | sedimentary |
| Polekhovskyite | MoNiP_2_ | 0.016 | metamorphic |
| Halamishite | Ni_5_P_4_ | 0.016 | metamorphic |
| Negevite | NiP_2_ | 0.016 | metamorphic |
| Transjordanite | Ni_2_P | 0.016 | metamorphic |
| Carpathite | C_24_H_12_ | 0.0053 | sedimentary |
| Simonellite | C_19_H_24_ | 0.0053 | sedimentary |
| Dinite | C_20_H_36_ | 0.0036 | sedimentary |
| Wampenite | C_18_H_16_ | 0.0018 | sedimentary |
| Phylloretine | C_18_H_18_ | 0.00013 | sedimentary |
| Kratochvilite | C_13_H_10_ | 0 | sedimentary |

**Table S4.** Soft acid-soft base minerals with low wMEE_CV_ values and maximum known ages at 1.108 billion years ago (Ga) that are located at Marathon Deposit, Coldwell Complex, Thunder Bay District, Ontario, Canada.

| Mineral | Chemical Formula | Maximum Age (Ga) | wMEE_CV_ | Locality |
| --- | --- | --- | --- | --- |
| Palladodymite | Pd_2_As | 1.108 | 0.005 | Marathon Deposit, Coldwell Complex, Thunder Bay District, Ontario, Canada |
| Cherepanovite | RhAs | 1.108 | 0.032 | Marathon Deposit, Coldwell Complex, Thunder Bay District, Ontario, Canada |
| Rhodarsenide | Rh_2_As | 1.108 | 0.026 | Marathon Deposit, Coldwell Complex, Thunder Bay District, Ontario, Canada |
| Marathonite | Pd_25_Ge_9_ | 1.108 | 0.04 | Marathon Deposit, Coldwell Complex, Thunder Bay District, Ontario, Canada |
| Palladogermanide | Pd_2_Ge | 1.108 | 0.051 | Marathon Deposit, Coldwell Complex, Thunder Bay District, Ontario, Canada |
| Skaergaardite | CuPd | 1.108 | 0.103 | Marathon Deposit, Coldwell Complex, Thunder Bay District, Ontario, Canada |
| Vasilite | (Pd,Cu)_16_(S,Te)_7_ | 1.108 | 0.106 | Marathon Deposit, Coldwell Complex, Thunder Bay District, Ontario, Canada |
| Coldwellite | Pd_3_Ag_2_S | 1.108 | 0.11 | Marathon Deposit, Coldwell Complex, Thunder Bay District, Ontario, Canada |
| Laflammeite | Pd_3_Pb_2_S_2_ | 1.108 | 0.073 | Marathon Deposit, Coldwell Complex, Thunder Bay District, Ontario, Canada |
|  |  |  |  |  |
|  |  |  |  |  |

**Fig. S1.** Full mineral chemistry network expansion through geologic time. Bipartite mineral chemistry network containing all mineral species and their constituent elements with minimum ages for the same localities that maximum known ages occur at in the time periods of (*A*) 4.0 to 4.7 Ga; (*B*) 2.5 to 4.7 Ga; (*C*) 0.5 to 4.7 Ga; (*D*) 0 to 4.7 Ga. Network lines (“edges”) connect minerals to all of their constituent elements. Element nodes are colored by Pauling scale electronegativity (Pauling, 1932) and mineral nodes are colored by weighted Mineral Element Electronegativity Coefficient of Variation (wMEE_CV_). Mineral nodes are sized by number of known localities. Figure created using dragon version 1.1.0 (<https://github.com/sjspielman/dragon>).

**Fig. S2.** Reduced number of soft acid/base mineral occurrences from 1.8 to 0.6 Ga. Weighted Mineral Element Electronegativity Coefficient of Variation (wMEE_CV_) plotted by the minimum age of >209,000 mineral occurrences in the Mineral Evolution Database in billions of years ago (Ga). Blue dots represent oxygen containing minerals and black dots represent non-oxygen containing minerals.

**Fig. S3.** (A) Box and whisker plot of weighted Mineral Element Electronegativity Coefficient of Variation (wMEE_CV_) values of mineral maximum known ages that occur in the time period 1 (4.34 Ga < t < 1.8 Ga), period 2 (1.8 Ga < t < 0.6 Ga), and period 3 (0.6 Ga < t < 0 Ga). (B) Box and whisker plot of weighted Mineral Element Electronegativity Coefficient of Variation (wMEE_CV_) values of >209,000 mineral occurrence ages that occur in the time periods 1, 2, and 3. (C) Bar plot of the ratio of the number of high wMEE_CV_ to low wMEE_CV_ minerals with maximum known ages that occur in time periods 1, 2, and 3. (D) Bar plot of the ratio of the number of high wMEE_CV_ to low wMEE_CV_ minerals with maximum known ages that occur in time periods 1, 2, and 3. High wMEE_CV_ values are >0.11 and low wMEE_CV_ values are <0.11.

**Supplementary Dataset. (separate file)** Calculated weighted Mineral Element Electronegativity Coefficient of Variation (wMEE_CV_) and weighted Mineral Element Electronegativity Mean (wMEEμ) values for all 4,579 unambiguous mineral formulas in the Mineral Evolution Database [MED; <https://rruff.info/evolution/>; (Golden, 2020); accessed February 3^rd^, 2020]. For any minerals which can interchangeably contain different elements, we assumed an equal proportion of those options. For example, the mineral urvantsevite, Pd(Bi,Pb)_2_, can interchangeably contain two of either bismuth (Bi) or lead (Pb) atoms. To tabulate the total number of elements, we would consider this formula as equivalent to: Pd(Bi_0.5_Pb_0.5_)_2_. Mineral formulas where average element contributions could not be calculated (ambiguous) were excluded.
